# Bis(2,6-diamino­pyridinium) hydrogen phthalate nitrate monohydrate

**DOI:** 10.1107/S1600536810004150

**Published:** 2010-02-06

**Authors:** Akbar Raissi Shabari, Maryam Safaeimovahed, Mehrdad Pourayoubi

**Affiliations:** a Faculty of Chemistry, Islamic Azad University-North Tehran Branch, Tehran, Iran; b Department of Chemistry, Ferdowsi University of Mashhad, Mashhad 91779, Iran

## Abstract

The title hydrated salt, 2C_5_H_8_N_3_^+^·C_8_H_5_O_4_^−^·NO_3_^−^·H_2_O, was obtained fortuitously from the reaction between 2,6-diamino­pyridine, phthalic acid and Co(NO_3_)_2_·6H_2_O at 343 K. The asymmetric unit consists of two crystallographically independent 2,6-diamino­pyridinium cations, a hydrogen phthalate anion, a nitrate ion and a water mol­ecule of crystallization which in the crystal structure are linked by inter­molecular O—H⋯O and N—H⋯O hydrogen bonds into a three-dimensional network. In the hydrogen phthalate anion, there is a very strong intra­molecular O—H⋯O hydrogen bond.

## Related literature

For a related structure, see: Al-Dajani *et al.* (2009 ▶). For a study of strong O—H⋯O hydrogen bonds, see: Gilli *et al.* (1994 ▶). 
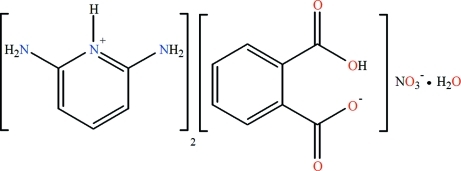


## Experimental

### 

#### Crystal data

2C_5_H_8_N_3_^+^·C_8_H_5_O_4_^−^·NO_3_^−^·H_2_O
*M* *_r_* = 465.43
Monoclinic, 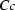

*a* = 3.6923 (3) Å
*b* = 37.857 (3) Å
*c* = 14.8415 (10) Å
β = 95.111 (2)°
*V* = 2066.3 (3) Å^3^
*Z* = 4
Mo *K*α radiation
μ = 0.12 mm^−1^
*T* = 120 K
0.29 × 0.26 × 0.22 mm

#### Data collection

Bruker SMART 1000 diffractometer
Absorption correction: multi-scan (*SADABS*; Sheldrick, 1996 ▶) *T*_min_ = 0.966, *T*_max_ = 0.974
15097 measured reflections
2737 independent reflections
2309 reflections with *I* > 2σ(*I*)
*R*_int_ = 0.034

#### Refinement

*R*[*F* ^2^ > 2σ(*F* ^2^)] = 0.046
*wR*(*F* ^2^) = 0.093
*S* = 1.01
2737 reflections
298 parameters
2 restraints
H-atom parameters constrained
Δρ_max_ = 0.26 e Å^−3^
Δρ_min_ = −0.19 e Å^−3^

### 

Data collection: *SMART* (Bruker, 1998 ▶); cell refinement: *SAINT-Plus* (Bruker, 1998 ▶); data reduction: *SAINT-Plus*; program(s) used to solve structure: *SHELXTL* (Sheldrick, 2008 ▶); program(s) used to refine structure: *SHELXTL*; molecular graphics: *SHELXTL*; software used to prepare material for publication: *SHELXTL*.

## Supplementary Material

Crystal structure: contains datablocks I, global. DOI: 10.1107/S1600536810004150/lh2987sup1.cif

Structure factors: contains datablocks I. DOI: 10.1107/S1600536810004150/lh2987Isup2.hkl

Additional supplementary materials:  crystallographic information; 3D view; checkCIF report

## Figures and Tables

**Table 1:** Hydrogen-bond geometry (Å, °)

*D*—H⋯*A*	*D*—H	H⋯*A*	*D*⋯*A*	*D*—H⋯*A*
O1—H1⋯O3	1.13	1.25	2.373 (4)	173
N1—H1*NA*⋯O1	0.90	2.10	2.950 (4)	157
N1—H1*NB*⋯O5	0.90	2.06	2.940 (4)	165
N2—H2*NA*⋯O1	0.90	2.48	3.259 (4)	146
N2—H2*NA*⋯O2	0.90	2.00	2.846 (3)	157
N3—H3*NB*⋯O5^i^	0.90	2.09	2.921 (4)	154
N3—H3*NA*⋯O2	0.90	2.25	3.074 (4)	153
N4—H4*NA*⋯O3^ii^	0.90	2.18	2.979 (4)	147
N4—H4*NB*⋯O6	0.90	2.02	2.891 (4)	163
N5—H5*NA*⋯O3^ii^	0.90	2.35	3.167 (4)	150
N5—H5*NA*⋯O4^ii^	0.90	2.07	2.889 (4)	150
N6—H6*NB*⋯O1*W*^iii^	0.90	1.98	2.825 (4)	157
N6—H6*NA*⋯O4^ii^	0.90	2.21	2.988 (4)	144
O1*W*—H1*WA*⋯O6^iv^	0.85	1.99	2.834 (4)	169
O1*W*—H1*WB*⋯O6	0.85	2.59	3.258 (4)	136
O1*W*—H1*WB*⋯O7	0.85	2.06	2.885 (3)	165

Symmetry codes: (i) 
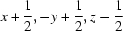
; (ii) 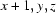; (iii) 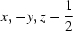; (iv) 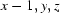.

**Fig. 1.**
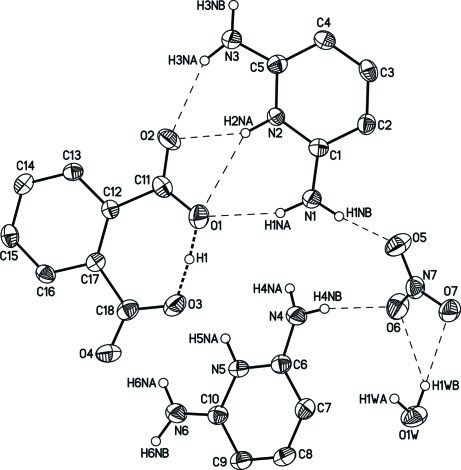
The asymmetric unit of the title hydrated salt, showing the atom-numbering scheme. Displacement ellipsoids are drawn at the 50 % probability level. The hydrogen bonds are shown by dashed lines.

**Fig. 2.**
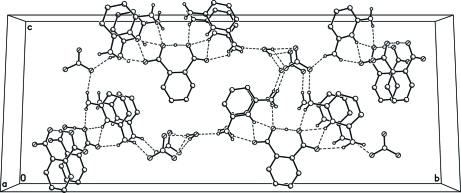
Part of the crystal structure of the title compound with hydrogen bonds shown as dashed lines.

## supplementary crystallographic information

### Comment

In previous work, the crystal structure of tetrakis(2,6-diaminopyridinium) diphthalate 2,6-diaminopyridine (Al-Dajani *et al.*, 2009) was investigated; we report here on the synthesis and crystal structure of a new proton-transfer salt of 2,6-diaminopyridine and phthalic acid. In the title compound (Fig. 1), phthalic acid is mono-deprotonated while the two 2,6-diaminopyridine components are protonated at the pyridine nitrogen atom. The two 2,6-diaminopyridinium cations are crystallographically independent. In the mono-anion, there is a very strong intramolecular [O—H···O]^-^ hydrogen bond (O1···O3 = 2.373 (4) Å) which is a result of the negative charge-assisted effect described by Gilli *et al.* (1994). The cations, anions and water molecules are liked into a 3-D network by O—H···O and N—H···O hydrogen bonds. A view of crystal packing is shown in Fig. 2.

### Experimental

The title compound was prepared according to the following procedure: A solution of phthalic acid (0.83 g, 5 mmol) in H_2_O (20 ml) was added to a solution of 2,6-diaminopyridine (0.545 g, 5 mmol) in H_2_O (5 ml) and stirred. To this solution, a solution of Co(NO_3_)_2_.6H_2_O (1.45 g, 5 mmol) in H_2_O (5 ml) was added and stirred at 343 K (20 minutes). The mixture was filtered and single crystals were obtained after slow evaporation at room temperature. IR (KBr, cm^-1^): 3436, 2347, 1650, 1385, 1053, 984, 773, 731, 485.

### Refinement

In the absence of significant anomalous dispersion effects the Friedel pairs were merged. H atoms were placed in calculated positions with C-H = 0.95, N-H = 0.90 and O-H = 0.84Å. The hydroxyl H atom of the hydrogen phthalate anion was included in an 'as found' position. All H atoms were included in the refinement in a riding-model approximation with U_iso_(H) = 1.2U_eq_(C,*N*) and 1.5U_eq_(O).

### Crystal data

**Table:**

2C_5_H_8_N_3_^+^·C_8_H_5_O_4_^−^·NO_3_^−^·H_2_O	*F*(000) = 976
*M**_r_* = 465.43	*D*_x_ = 1.496 Mg m^−^^3^
Monoclinic, *C**c*	Mo *K*α radiation, λ = 0.71073 Å
Hall symbol: C -2yc	Cell parameters from 5008 reflections
*a* = 3.6923 (3) Å	θ = 2.2–27.2°
*b* = 37.857 (3) Å	µ = 0.12 mm^−^^1^
*c* = 14.8415 (10) Å	*T* = 120 K
β = 95.111 (2)°	Prism, colorless
*V* = 2066.3 (3) Å^3^	0.29 × 0.26 × 0.22 mm
*Z* = 4	

### Data collection

**Table:**

Bruker SMART 1000 diffractometer	2737 independent reflections
Radiation source: fine-focus sealed tube	2309 reflections with *I* > 2σ(*I*)
graphite	*R*_int_ = 0.034
φ and ω scans	θ_max_ = 29.0°, θ_min_ = 1.8°
Absorption correction: multi-scan (*SADABS*; Sheldrick, 1996)	*h* = −5→5
*T*_min_ = 0.966, *T*_max_ = 0.974	*k* = −50→49
15097 measured reflections	*l* = −20→20

### Refinement

**Table:**

Refinement on *F*^2^	Primary atom site location: structure-invariant direct methods
Least-squares matrix: full	Secondary atom site location: difference Fourier map
*R*[*F*^2^ > 2σ(*F*^2^)] = 0.046	Hydrogen site location: mixed
*wR*(*F*^2^) = 0.093	H-atom parameters constrained
*S* = 1.00	*w* = 1/[σ^2^(*F*_o_^2^) + (0.02*P*)^2^ + 3.5*P*] where *P* = (*F*_o_^2^ + 2*F*_c_^2^)/3
2737 reflections	(Δ/σ)_max_ < 0.001
298 parameters	Δρ_max_ = 0.26 e Å^−^^3^
2 restraints	Δρ_min_ = −0.19 e Å^−^^3^

### Special details

**Table:**

Geometry. All esds (except the esd in the dihedral angle between two l.s. planes) are estimated using the full covariance matrix. The cell esds are taken into account individually in the estimation of esds in distances, angles and torsion angles; correlations between esds in cell parameters are only used when they are defined by crystal symmetry. An approximate (isotropic) treatment of cell esds is used for estimating esds involving l.s. planes.
Refinement. Refinement of *F*^2^ against ALL reflections. The weighted *R*-factor wR and goodness of fit *S* are based on *F*^2^, conventional *R*-factors *R* are based on *F*, with *F* set to zero for negative *F*^2^. The threshold expression of *F*^2^ > σ(*F*^2^) is used only for calculating *R*-factors(gt) etc. and is not relevant to the choice of reflections for refinement. *R*-factors based on *F*^2^ are statistically about twice as large as those based on *F*, and *R*- factors based on ALL data will be even larger.

### Fractional atomic coordinates and isotropic or equivalent isotropic displacement parameters (Å^2^)

**Table:**

	*x*	*y*	*z*	*U*_iso_*/*U*_eq_	
N1	0.6231 (8)	0.17540 (6)	0.49397 (18)	0.0283 (6)	
H1NA	0.4475	0.1658	0.4557	0.034*	
H1NB	0.6041	0.1721	0.5534	0.034*	
N2	0.6881 (7)	0.22109 (6)	0.39298 (16)	0.0229 (5)	
H2NA	0.5601	0.2072	0.3525	0.027*	
N3	0.7201 (9)	0.26246 (7)	0.28055 (19)	0.0355 (7)	
H3NB	0.7787	0.2848	0.2672	0.043*	
H3NA	0.6032	0.2454	0.2473	0.043*	
C1	0.7408 (8)	0.20826 (8)	0.4790 (2)	0.0229 (6)	
C2	0.9210 (9)	0.22967 (8)	0.5452 (2)	0.0268 (6)	
H2A	0.9646	0.2217	0.6059	0.032*	
C3	1.0348 (8)	0.26286 (8)	0.5202 (2)	0.0274 (6)	
H3A	1.1589	0.2775	0.5649	0.033*	
C4	0.9751 (8)	0.27550 (8)	0.4329 (2)	0.0269 (6)	
H4A	1.0539	0.2984	0.4177	0.032*	
C5	0.7960 (8)	0.25365 (8)	0.3679 (2)	0.0253 (6)	
N4	0.5267 (8)	0.09027 (7)	0.51218 (19)	0.0296 (6)	
H4NA	0.6869	0.0988	0.4753	0.036*	
H4NB	0.5352	0.1036	0.5625	0.036*	
N5	0.4276 (7)	0.03920 (7)	0.42957 (18)	0.0258 (5)	
H5NA	0.5180	0.0520	0.3857	0.031*	
N6	0.3504 (8)	−0.00825 (7)	0.33294 (19)	0.0331 (6)	
H6NB	0.2189	−0.0275	0.3158	0.040*	
H6NA	0.4457	0.0043	0.2893	0.040*	
C6	0.4164 (8)	0.05637 (8)	0.5096 (2)	0.0251 (6)	
C7	0.2886 (9)	0.03842 (8)	0.5820 (2)	0.0283 (6)	
H7A	0.2777	0.0497	0.6389	0.034*	
C8	0.1770 (8)	0.00351 (9)	0.5693 (2)	0.0295 (7)	
H8A	0.0886	−0.0090	0.6184	0.035*	
C9	0.1914 (9)	−0.01353 (9)	0.4870 (2)	0.0294 (7)	
H9A	0.1147	−0.0374	0.4797	0.035*	
C10	0.3200 (8)	0.00492 (8)	0.4155 (2)	0.0255 (6)	
O1	0.1534 (7)	0.15535 (7)	0.33076 (16)	0.0377 (6)	
H1	0.0380	0.1279	0.3375	0.057*	
O2	0.3398 (7)	0.19199 (6)	0.23096 (17)	0.0367 (6)	
O3	−0.1237 (7)	0.09909 (7)	0.34030 (16)	0.0388 (6)	
O4	−0.3587 (7)	0.05715 (7)	0.25269 (18)	0.0429 (6)	
C11	0.1856 (8)	0.16417 (8)	0.2484 (2)	0.0269 (6)	
C12	0.0332 (7)	0.14044 (8)	0.1715 (2)	0.0204 (6)	
C13	0.0674 (8)	0.15452 (8)	0.0853 (2)	0.0232 (6)	
H13A	0.1735	0.1773	0.0811	0.028*	
C14	−0.0452 (8)	0.13698 (8)	0.0067 (2)	0.0263 (6)	
H14A	−0.0135	0.1472	−0.0505	0.032*	
C15	−0.2063 (8)	0.10402 (8)	0.0126 (2)	0.0273 (6)	
H15A	−0.2905	0.0916	−0.0409	0.033*	
C16	−0.2436 (8)	0.08934 (8)	0.0964 (2)	0.0248 (6)	
H16A	−0.3498	0.0666	0.0994	0.030*	
C17	−0.1311 (8)	0.10676 (8)	0.1768 (2)	0.0225 (6)	
C18	−0.2081 (9)	0.08613 (9)	0.2609 (2)	0.0295 (7)	
N7	0.5255 (8)	0.14787 (7)	0.72886 (18)	0.0307 (6)	
O5	0.4868 (8)	0.17644 (6)	0.68613 (17)	0.0419 (6)	
O6	0.6450 (7)	0.12132 (6)	0.69036 (16)	0.0392 (6)	
O7	0.4512 (7)	0.14595 (6)	0.80893 (16)	0.0380 (6)	
O1W	0.0932 (8)	0.07824 (6)	0.81011 (18)	0.0400 (6)	
H1WA	−0.0387	0.0932	0.7797	0.060*	
H1WB	0.2333	0.0959	0.8075	0.060*	

### Atomic displacement parameters (Å^2^)

**Table:**

	*U*^11^	*U*^22^	*U*^33^	*U*^12^	*U*^13^	*U*^23^
N1	0.0383 (15)	0.0226 (13)	0.0237 (13)	−0.0027 (11)	0.0010 (11)	0.0000 (10)
N2	0.0276 (13)	0.0200 (12)	0.0211 (12)	−0.0015 (10)	0.0019 (10)	−0.0023 (10)
N3	0.0520 (18)	0.0264 (14)	0.0270 (14)	−0.0091 (13)	−0.0020 (13)	0.0015 (11)
C1	0.0223 (14)	0.0206 (14)	0.0261 (15)	0.0022 (11)	0.0042 (11)	−0.0003 (11)
C2	0.0310 (16)	0.0269 (15)	0.0221 (14)	0.0018 (13)	−0.0002 (12)	−0.0017 (12)
C3	0.0277 (16)	0.0256 (15)	0.0285 (16)	0.0016 (12)	−0.0001 (12)	−0.0091 (12)
C4	0.0264 (15)	0.0223 (15)	0.0324 (16)	−0.0007 (12)	0.0044 (12)	−0.0023 (13)
C5	0.0285 (15)	0.0226 (15)	0.0254 (15)	0.0014 (12)	0.0056 (12)	0.0002 (12)
N4	0.0385 (15)	0.0230 (13)	0.0276 (13)	−0.0039 (11)	0.0042 (11)	−0.0019 (10)
N5	0.0293 (13)	0.0210 (12)	0.0273 (13)	−0.0020 (10)	0.0030 (10)	0.0032 (10)
N6	0.0440 (17)	0.0260 (14)	0.0295 (14)	−0.0065 (12)	0.0047 (12)	−0.0026 (11)
C6	0.0248 (15)	0.0225 (14)	0.0282 (15)	0.0034 (11)	0.0025 (12)	0.0022 (12)
C7	0.0304 (16)	0.0299 (16)	0.0247 (15)	0.0028 (13)	0.0038 (12)	0.0012 (13)
C8	0.0273 (16)	0.0299 (17)	0.0318 (16)	0.0018 (13)	0.0053 (13)	0.0099 (13)
C9	0.0306 (17)	0.0255 (15)	0.0322 (16)	−0.0006 (13)	0.0035 (13)	0.0053 (13)
C10	0.0239 (14)	0.0231 (15)	0.0292 (16)	0.0027 (12)	0.0016 (11)	0.0030 (12)
O1	0.0485 (15)	0.0380 (13)	0.0262 (12)	−0.0022 (11)	0.0012 (10)	−0.0044 (10)
O2	0.0404 (14)	0.0301 (12)	0.0397 (14)	−0.0112 (10)	0.0042 (11)	−0.0086 (10)
O3	0.0516 (16)	0.0393 (14)	0.0259 (12)	−0.0030 (12)	0.0065 (11)	0.0062 (11)
O4	0.0471 (15)	0.0370 (14)	0.0444 (15)	−0.0143 (12)	0.0021 (12)	0.0138 (12)
C11	0.0233 (15)	0.0267 (15)	0.0307 (16)	0.0016 (12)	0.0028 (12)	−0.0067 (13)
C12	0.0178 (13)	0.0211 (13)	0.0224 (13)	0.0033 (10)	0.0010 (10)	−0.0017 (11)
C13	0.0225 (14)	0.0206 (13)	0.0269 (14)	0.0023 (11)	0.0040 (11)	0.0025 (12)
C14	0.0276 (16)	0.0293 (15)	0.0224 (14)	0.0060 (12)	0.0039 (12)	0.0036 (12)
C15	0.0258 (15)	0.0299 (16)	0.0257 (15)	0.0043 (12)	−0.0012 (12)	−0.0067 (12)
C16	0.0226 (15)	0.0195 (13)	0.0316 (16)	0.0001 (11)	−0.0014 (12)	−0.0008 (12)
C17	0.0212 (14)	0.0238 (14)	0.0232 (14)	0.0028 (11)	0.0049 (11)	0.0007 (11)
C18	0.0257 (16)	0.0310 (16)	0.0318 (16)	0.0052 (13)	0.0021 (12)	0.0072 (13)
N7	0.0372 (16)	0.0307 (14)	0.0239 (14)	0.0012 (12)	0.0004 (11)	−0.0044 (11)
O5	0.0644 (18)	0.0302 (13)	0.0324 (13)	0.0097 (12)	0.0118 (12)	0.0070 (10)
O6	0.0564 (16)	0.0311 (13)	0.0299 (13)	0.0120 (11)	0.0019 (11)	−0.0063 (10)
O7	0.0551 (17)	0.0352 (14)	0.0243 (11)	−0.0027 (12)	0.0071 (11)	−0.0020 (10)
O1W	0.0516 (16)	0.0255 (12)	0.0431 (14)	0.0061 (11)	0.0053 (12)	0.0075 (10)

### Geometric parameters (Å, °)

**Table:**

N1—C1	1.343 (4)	C8—C9	1.386 (5)
N1—H1NA	0.8999	C8—H8A	0.9500
N1—H1NB	0.9001	C9—C10	1.390 (4)
N2—C5	1.358 (4)	C9—H9A	0.9500
N2—C1	1.364 (4)	O1—C11	1.283 (4)
N2—H2NA	0.9000	O1—H1	1.1299
N3—C5	1.343 (4)	O2—C11	1.235 (4)
N3—H3NB	0.8998	O3—C18	1.289 (4)
N3—H3NA	0.8998	O3—H1	1.2468
C1—C2	1.396 (4)	O4—C18	1.231 (4)
C2—C3	1.386 (4)	C11—C12	1.520 (4)
C2—H2A	0.9500	C12—C13	1.402 (4)
C3—C4	1.379 (4)	C12—C17	1.417 (4)
C3—H3A	0.9500	C13—C14	1.374 (4)
C4—C5	1.393 (4)	C13—H13A	0.9500
C4—H4A	0.9500	C14—C15	1.389 (5)
N4—C6	1.346 (4)	C14—H14A	0.9500
N4—H4NA	0.9000	C15—C16	1.381 (4)
N4—H4NB	0.9001	C15—H15A	0.9500
N5—C6	1.358 (4)	C16—C17	1.393 (4)
N5—C10	1.368 (4)	C16—H16A	0.9500
N5—H5NA	0.9000	C17—C18	1.520 (4)
N6—C10	1.336 (4)	N7—O7	1.245 (3)
N6—H6NB	0.8996	N7—O6	1.255 (3)
N6—H6NA	0.8999	N7—O5	1.256 (4)
C6—C7	1.389 (4)	O1W—H1WA	0.8497
C7—C8	1.392 (5)	O1W—H1WB	0.8496
C7—H7A	0.9500		
			
C1—N1—H1NA	119.7	C9—C8—C7	121.8 (3)
C1—N1—H1NB	110.2	C9—C8—H8A	119.1
H1NA—N1—H1NB	116.7	C7—C8—H8A	119.1
C5—N2—C1	124.0 (3)	C8—C9—C10	118.8 (3)
C5—N2—H2NA	119.7	C8—C9—H9A	120.6
C1—N2—H2NA	116.2	C10—C9—H9A	120.6
C5—N3—H3NB	114.3	N6—C10—N5	116.6 (3)
C5—N3—H3NA	113.5	N6—C10—C9	125.1 (3)
H3NB—N3—H3NA	131.9	N5—C10—C9	118.3 (3)
N1—C1—N2	117.6 (3)	C11—O1—H1	113.1
N1—C1—C2	124.3 (3)	C18—O3—H1	112.4
N2—C1—C2	118.1 (3)	O2—C11—O1	120.4 (3)
C3—C2—C1	118.4 (3)	O2—C11—C12	119.5 (3)
C3—C2—H2A	120.8	O1—C11—C12	120.1 (3)
C1—C2—H2A	120.8	C13—C12—C17	117.8 (3)
C4—C3—C2	122.6 (3)	C13—C12—C11	113.8 (3)
C4—C3—H3A	118.7	C17—C12—C11	128.5 (3)
C2—C3—H3A	118.7	C14—C13—C12	123.1 (3)
C3—C4—C5	118.1 (3)	C14—C13—H13A	118.5
C3—C4—H4A	120.9	C12—C13—H13A	118.5
C5—C4—H4A	120.9	C13—C14—C15	118.7 (3)
N3—C5—N2	116.7 (3)	C13—C14—H14A	120.7
N3—C5—C4	124.5 (3)	C15—C14—H14A	120.7
N2—C5—C4	118.8 (3)	C16—C15—C14	119.7 (3)
C6—N4—H4NA	122.5	C16—C15—H15A	120.1
C6—N4—H4NB	123.2	C14—C15—H15A	120.1
H4NA—N4—H4NB	109.1	C15—C16—C17	122.3 (3)
C6—N5—C10	123.8 (3)	C15—C16—H16A	118.8
C6—N5—H5NA	114.7	C17—C16—H16A	118.8
C10—N5—H5NA	121.5	C16—C17—C12	118.4 (3)
C10—N6—H6NB	118.3	C16—C17—C18	113.4 (3)
C10—N6—H6NA	122.2	C12—C17—C18	128.3 (3)
H6NB—N6—H6NA	117.4	O4—C18—O3	120.0 (3)
N4—C6—N5	116.7 (3)	O4—C18—C17	119.5 (3)
N4—C6—C7	124.5 (3)	O3—C18—C17	120.5 (3)
N5—C6—C7	118.8 (3)	O7—N7—O6	120.3 (3)
C6—C7—C8	118.5 (3)	O7—N7—O5	120.5 (3)
C6—C7—H7A	120.8	O6—N7—O5	119.2 (3)
C8—C7—H7A	120.8	H1WA—O1W—H1WB	76.9
			
C5—N2—C1—N1	−179.3 (3)	O2—C11—C12—C13	−3.8 (4)
C5—N2—C1—C2	−0.9 (4)	O1—C11—C12—C13	176.1 (3)
N1—C1—C2—C3	178.7 (3)	O2—C11—C12—C17	175.6 (3)
N2—C1—C2—C3	0.4 (4)	O1—C11—C12—C17	−4.5 (5)
C1—C2—C3—C4	0.4 (5)	C17—C12—C13—C14	−1.0 (4)
C2—C3—C4—C5	−0.6 (5)	C11—C12—C13—C14	178.5 (3)
C1—N2—C5—N3	−179.3 (3)	C12—C13—C14—C15	1.1 (4)
C1—N2—C5—C4	0.7 (4)	C13—C14—C15—C16	−1.2 (4)
C3—C4—C5—N3	−179.9 (3)	C14—C15—C16—C17	1.3 (5)
C3—C4—C5—N2	0.1 (4)	C15—C16—C17—C12	−1.2 (4)
C10—N5—C6—N4	−179.0 (3)	C15—C16—C17—C18	178.4 (3)
C10—N5—C6—C7	0.0 (4)	C13—C12—C17—C16	1.0 (4)
N4—C6—C7—C8	178.8 (3)	C11—C12—C17—C16	−178.4 (3)
N5—C6—C7—C8	−0.1 (4)	C13—C12—C17—C18	−178.5 (3)
C6—C7—C8—C9	0.2 (5)	C11—C12—C17—C18	2.1 (5)
C7—C8—C9—C10	−0.2 (5)	C16—C17—C18—O4	−0.5 (4)
C6—N5—C10—N6	−179.5 (3)	C12—C17—C18—O4	179.0 (3)
C6—N5—C10—C9	0.0 (4)	C16—C17—C18—O3	−179.1 (3)
C8—C9—C10—N6	179.6 (3)	C12—C17—C18—O3	0.4 (5)
C8—C9—C10—N5	0.1 (4)		

### Hydrogen-bond geometry (Å, °)

**Table:**

*D*—H···*A*	*D*—H	H···*A*	*D*···*A*	*D*—H···*A*
O1—H1···O3	1.13	1.25	2.373 (4)	173
N1—H1NA···O1	0.90	2.10	2.950 (4)	157
N1—H1NB···O5	0.90	2.06	2.940 (4)	165
N2—H2NA···O1	0.90	2.48	3.259 (4)	146
N2—H2NA···O2	0.90	2.00	2.846 (3)	157
N3—H3NB···O5^i^	0.90	2.09	2.921 (4)	154
N3—H3NA···O2	0.90	2.25	3.074 (4)	153
N4—H4NA···O3^ii^	0.90	2.18	2.979 (4)	147
N4—H4NB···O6	0.90	2.02	2.891 (4)	163
N5—H5NA···O3^ii^	0.90	2.35	3.167 (4)	150
N5—H5NA···O4^ii^	0.90	2.07	2.889 (4)	150
N6—H6NB···O1W^iii^	0.90	1.98	2.825 (4)	157
N6—H6NA···O4^ii^	0.90	2.21	2.988 (4)	144
O1W—H1WA···O6^iv^	0.85	1.99	2.834 (4)	169
O1W—H1WB···O6	0.85	2.59	3.258 (4)	136
O1W—H1WB···O7	0.85	2.06	2.885 (3)	165

Symmetry codes: (i) *x*+1/2, −*y*+1/2, *z*−1/2; (ii) *x*+1, *y*, *z*; (iii) *x*, −*y*, *z*−1/2; (iv) *x*−1, *y*, *z*.
